# Reincarnation of Bacteriocins From the *Lactobacillus* Pangenomic Graveyard

**DOI:** 10.3389/fmicb.2018.01298

**Published:** 2018-07-02

**Authors:** Fergus W. J. Collins, Beatriz Mesa-Pereira, Paula M. O'Connor, Mary C. Rea, Colin Hill, R. Paul Ross

**Affiliations:** ^1^Teagasc Food Research Centre, Cork, Ireland; ^2^APC Microbiome Ireland, University College Cork, Cork, Ireland; ^3^Department of Microbiology, University College Cork, Cork, Ireland

**Keywords:** bacteriocins, pediocin, heterologous expression, *Escherichia coli*, *Lactobacillus*

## Abstract

Bacteria commonly produce narrow spectrum bacteriocins as a means of inhibiting closely related species competing for similar resources in an environment. The increasing availability of genomic data means that it is becoming easier to identify bacteriocins encoded within genomes. Often, however, the presence of bacteriocin genes in a strain does not always translate into biological antimicrobial activity. For example, when analysing the *Lactobacillus* pangenome we identified strains encoding ten pediocin-like bacteriocin structural genes which failed to display inhibitory activity. Nine of these bacteriocins were novel whilst one was identified as the previously characterized bacteriocin “penocin A.” The composition of these bacteriocin operons varied between strains, often with key components missing which are required for bacteriocin production, such as dedicated bacteriocin transporters and accessory proteins. In an effort to functionally express these bacteriocins, the structural genes for the ten pediocin homologs were cloned alongside the dedicated pediocin PA-1 transporter in both *Escherichia coli* and *Lactobacillus paracasei* heterologous hosts. Each bacteriocin was cloned with its native leader sequence and as a fusion protein with the pediocin PA-1 leader sequence. Several of these bacteriocins displayed a broader spectrum of inhibition than the original pediocin PA-1. We show how potentially valuable bacteriocins can easily be “reincarnated” from *in silico* data and produced *in vitro* despite often lacking the necessary accompanying machinery. Moreover, the study demonstrates how genomic datasets such as the *Lactobacilus* pangenome harbor a potential “arsenal” of antimicrobial activity with the possibility of being activated when expressed in more genetically amenable hosts.

## Introduction

Bacteria exist in complex communities, under constant competition from other strains and species for nutrients and space. The production of antimicrobial peptides known as bacteriocins has been shown to be one means by which such strains can gain a competitive advantage (Kommineni et al., 2015). Bacteriocins can be broad spectrum, inhibiting a variety of bacteria, or narrow spectrum where they inhibit primarily closely related species. As closely related species are more likely to occupy the same environmental niche as the producer, these bacteriocin genes could offer a potentially useful armory of antimicrobials in helping the producer establish itself in such a niche.

The increasing availability of genomic data has changed the way we identify and study bacteriocins in communities. Bioinformatic screening tools such as BAGEL (de Jong et al., 2010) and antiSMASH (Weber et al., 2015) can now process vast amounts of genomic data to search for antimicrobial operons and genes (Letzel et al., 2014; Walsh et al., 2015). This allows us to identify previously uncharacterized bacteriocins and antibiotics, and to understand the extent of which strains encode these natural weapons for targeting competitors. In many of these cases however, the bacteriocin genes appear to be inactive antimicrobial relics which are unlikely to play an active role, given the degradation of the surrounding accessory genes. Mutations within genes, loss of key genes within operons and tight transcriptional regulation can all prevent cells from producing these antimicrobials. This means that many such strains harbor potentially useful bacteriocins that are destined to remain uncharacterized due to a lack of *in vitro* production. One method to overcome this issue is to clone the antimicrobial operon into a host where expression can be controlled. This allows for the natural bacteriocin regulation to be circumvented and/or gene loss to be overcome, thus ensuring production of otherwise unavailable antimicrobials for further characterization and potential exploitation.

In this study we focused on the expression of potential class IIa bacteriocins from members of the *Lactobacillus* Genus Complex. Bacteriocins are divided into different classes based on their modifications, structure and mode of action. Class II bacteriocins are not subject to extensive post-translational modification and the class IIa bacteriocins are single peptides with a highly conserved “YGNGV” N-terminal sequence. These bacteriocins (also known as “pediocin-like” bacteriocins) are relatively narrow spectrum and display high levels of activity against *Listeria* species (Cotter et al., 2005). They bind to the mannose phosphotransferase transport system in sensitive strains and subsequently induce pore formation which leads to cell death (Zhou et al., 2016). The effective production of these bacteriocins depends on the production of several other associated proteins (Fimland et al., 2005). For example, an associated ABC transporter must be produced by cells in order to transport the bacteriocin outside the cell, and an immunity protein is also required to protect the producing strain from being killed by its own bacteriocin (Drider et al., 2006). Certain class IIa bacteriocins also require an accessory protein for correct disulphide bond formation (Oppegård et al., 2015). These operons may also have a three-component regulatory system which regulates expression of the bacteriocin. Here, when the concentration of an inducer peptide encoded within the operon reaches a certain level it signals a transmembrane histidine kinase which in turn activates a response regulator which initiates transcription of the bacteriocin operon (Ennahar et al., 2000).

The *Lactobacillus* Complex analyzed in this study encompasses the *Lactobacillus, Pediococcus, Leuconostoc, Weissella, Fructobacillus*, and *Oenococcus* genera and numerous pediocin-like bacteriocins have been associated with this grouping (Collins et al., 2017). Previously Sun et al. (2015) sequenced the genomes of 213 strains belonging to this complex, these were then subject to both *in silico* and *in vitro* bacteriocin screening (Collins et al., 2017). Numerous strains from that study were found to encode bacteriocin operons but did not show *in vitro* bacteriocin production. Several of these strains encoded complete operons for pediocin-like bacteriocins, whilst more had incomplete operons harboring just structural and immunity genes. Whereas regulatory issues may prevent expression of bacteriocins in strains harboring complete operons, those without the necessary transporter systems seem unlikely to be produced. It is unclear as to why such strains encode intact bacteriocin and immunity genes whilst lacking the machinery needed to actually produce and secrete these antimicrobials. One potential explanation for this is that these strains may have maintained the bacteriocin immunity gene under selective pressure from these antimicrobials. The neighboring bacteriocin encoding gene is small and may have simply been maintained due its proximity to the immunity gene, the much larger transport machinery may then have been lost.

In this study we use an expression systems derived by Mesa-Pereira et al. (2017) to reincarnate these bacteriocins, allowing for *in vitro* production. It was found that for production of pediocin PA-1, only the structural gene and the transporter were necessary for bacteriocin production and secretion. As some of our putative novel bacteriocins lacked an associated transporter, the pediocin PA-1 transporter was used in each case. Bacteriocin peptides were detected by mass spectrometry and activity was identified by screening against a range of indicator organisms. The systems used have allowed us to isolate seven novel bacteriocins and represents a unique and rapid way of producing bacteriocins. This method allows us to reincarnate otherwise ineffectual antimicrobial relics identified solely by *in silico* methods.

## Materials and methods

### Strains and culture conditions

Bacterial strains and growth conditions used are displayed in Supplementary Table 2. *E. coli* HST08 Stellar^TM^ cells (Takara BIO USA, Inc., Mountain View, CA) were used for normal cloning methods, *E. coli* BL21 Tuner^TM^ (DE3) cells (Novagen, EMD Millipore, Billerica, MA) were used for expression of the transformed genes. *E. coli* strains were grown in Luria-Bertani (LB) media containing 50 μg/ml of ampicillin for plasmid selection. IPTG (Isopropyl β-D-1-thiogalactopyranoside) (Fisher Scientific, Dublin, Ireland) was added to the growth media to induce gene expression. L-(+)-arabinose and D -(+)-glucose (Sigma Aldrich, Arklow, Ireland) were also added to the media to control plasmid levels in recombinant cells. *L. paracasei* NFBC338 cells were grown in modified MRS media containing 0.05% cysteine and 10 μg/ml chloramphenicol (Sigma Aldrich, Arklow, Ireland).

### Molecular cloning and gene expression

Total genomic DNA was extracted from the bacteriocin encoding strains using the GenElute Bacterial Genomic DNA Kit (Sigma Aldrich, Arklow, Ireland). Primers were designed for amplification of the bacteriocins and transporter genes as outlined in the In-Fusion HD cloning protocol (Takara BIO USA, Inc., Mountain View, CA). For amplification of genes containing the bacteriocin along with the native leader sequence, the genes were amplified by PCR from the original genomic DNA of the host strain using CloneAmp Hifi PCR premix (Takara BIO USA, Inc., Mountain View, CA). Recursive PCR was used for the synthesis of fusion genes containing the pediocin PA-1 leader sequence joined to the bacteriocin structural gene (Prodromou and Pearl, 1992). In both cases, the fragment containing the transporter encoded gene (*pedD)* was amplified using pMPB1 pedD Fw1 and pMPB1 pedD Rv1 using *P. acidilactici* LMG2351 as a DNA template. The oligonucleotides used in this study are listed in Supplementary Table 3. PCR products were purified using the Illustra GFX PCR DNA and Gel Band Purificatoin kit (GE Healthcare, UK), these were then inserted into the linearized SphI and AvrII pMPB1 vector using In-Fusion HD cloning Plus. pMPB1 vector is based on the commercial plasmid pETcoco^TM^-2 which allows a dual control of expression; at transcriptional level by IPTG induction and for amplification at the DNA level replication by L-arabinose (Sektas and Szybalski, 2002).

The constructions were transformed into Stellar^TM^ competent cells and colonies were selected on LB agar plates containing 50 μg/ml of ampicillin and 0.2% D-glucose. The transformants were confirmed by colony PCR reactions. L-arabinose was used to increase the plasmid copy number in cells and plasmid DNA was isolated using the NucleoSpin plasmid kit (Macherey-Nagel, Duren, Germany) to be analyzed subsequently by double digestion and sequencing. For bacteriocin expression, Tuner^TM^ (DE3) *E. coli* cells were transformed with the bacteriocin encoding vectors of interest. The transformants were then grown in LB containing 50 μg/ml of ampicillin and 0.2% glucose which maintains the plasmid in a single copy state. Once cells had grown to an OD_600_ of 0.5–0.7, IPTG was added at a concentration of 50 μM to induce expression of the bacteriocin genes.

For expression in *L. paracasei* NFBC338 the pNZ44 plasmid was used which contains the p44 constitutive promoter. Each bacteriocin was cloned containing the pediocin promoter and the pedD transporter, PCR fragments were amplified from positive Stellar^TM^ transformants using the pNZ44 pedA FW and pNZ44 pedD RV oligonucleotides. These were inserted into the linearized NcoI-HindIII pNZ44 vector using In-Fusion HD cloning Plus. Constructions were cloned into Stellar^TM^ competent cells and confirmed as before. For expression, *L. paracasei* NFBC338 cells were transformed with the bacteriocin-containing plasmids by electroporation. Transformants were grown overnight in MRS containing 0.05% cysteine and 10 μg/ml of chloramphenicol.

### Bacteriocin assays

Bacteriocin activity was determined using well diffusion assays (WDAs). For Tuner^TM^ (DE3) *E. coli* transformants both the cell supernatant and the cell lysates were analyzed for antimicrobial activity, whilst only the supernatants were analyzed for *L. paracasei* NFBC338 cells. Supernatant was isolated by centrifugation of the liquid cultures at 4000 RCF for 15 min, these supernatants were then filtered using 0.20 μm membrane filters. For cell lysates, cell pellets were resuspended in 5 ml of phosphate-buffered saline (PBS), cells were lysed by sonication using a MSE Soniprep 150 (MSE, London, UK). Lysates were subsequently centrifuged at 4000 RCF for 15 min and the resulting supernatants were filtered using 0.20 μm membrane filters. For the indicator plates, 50 μl of an overnight culture of the indicator strain was added to the appropriate media containing 1% agar. Plates were cooled and 7 mm wells were bored in the agar. 50 μl of the cell supernatants/lysates being tested were added to each well and plates were refrigerated for 2 h prior to incubation under the appropriate conditions. WDAs were carried out in triplicate for each bacteriocin.

### Bacteriocin purification and mass spectrometry

Purification and analysis was carried out for bacteriocins encoding the pediocin PA-1 leader sequence from Tuner^TM^ (DE3) *E. coli* transformants. 85 ml of each culture supernatant was applied to 2 ml SP sepharose columns (GE Healthcare, UK) pre-equilibrated with 25 ml 20 mM potassium phosphate buffer 25% acetonitrile, pH 2.5. Columns were washed with 20 ml 20 mM potassium phosphate buffer 25% acetonitrile, pH 2.5, the bacteriocins analyzed eluted from columns with 25 ml 20 mM potassium phosphate buffer 25% acetonitrile containing 2 M KCl, pH 2.5. Eluents were passed through a 6 ml, 500 mg Strata–E C18 SPE column pre-equilibrated (Phenomenex, Cheshire, UK) with methanol and water. The column was washed with 6 ml 30% ethanol and then 6 ml 70% 2-propanol 0.1 TFA (IPA). MALDI TOF colony mass spectroscopy was carried out on the eluents using an An Axima TOF^2^plus MALDI TOF mass spectrometer (Shimadzu Biotech, Manchester, UK) in positive-ion reflectron mode.

## Results

### Identification of “silent” pediocin homologs

Previous work performed by our group analyzed bacteriocin production in 213 *Lactobacillus* and related species (Collins et al., 2017). *In silico* screening identified the presence of bacteriocin operons (or remnants of operons) encoded within the genome of a large number of these strains. Strains encoding bacteriocins were then tested for *in vitro* bacteriocin production but several strains failed to display antimicrobial activity (Sun et al., 2015; Collins et al., 2017). This study focused on the class IIa bacteriocins; eight strains were found to encode ten of these pediocin-like bacteriocins but failed to display any antimicrobial production. Of these ten potential bacteriocins identified, nine were novel and one was identified as penocin A, a bacteriocin previously characterized from *P. pentosaceus* ATCC 25745 (Diep et al., 2006; Table 1).

**Table 1:** Bacteriocins analyzed and percentage identity to pediocin PA-1.

**Encoding strain**	**Bacteriocin**	**Leader**	**Structural gene**	**Structural gene % amino acid identify to pediocin PA-1**
*P. acidilactici* LMG 2351	Pediocin PA-1 (PedA)	————MKKIEKLTEKEMANIIGG	–KYYGNGVTCG-KHSCSVDWGKATTCIINNGAMAWATGGHQGNHKC—–	–
*P. pentosaceus* DSM 20336	Pediocin 20336a (Ped20336)	————MKKIEKLTEKEMANIIGG	–KYYGNGLYCG-KHSCSVDWGKATTCIINNGAMAWATGGHQGTHKC——	93.18
*L. rennini* DSM 20253	Rennicin B (RenB)	——————-MLSKEELTQVNGG	–KYYGNGVSCG-KHSCSVDWGKATTCTINNSAMAWATSGHQGNHKC—-	90.91
*L. rennini* DSM 20253	Rennicin A (RenA)	——————-MLSKEELTQVNGG	–KYYGNGVSCS-KHSCSVDWGKALTCTINNGAMAWTTGGHQGNHKC—-	88.64
*L. hordei* DSM 19519	Hordeiocin (Hrd)	————-MKKEIELSEKELVRIIGG	–KYYGNGVSCTKKHGCKVNWGQAFTCSVNRFANFGH–G————-NC—	56.76
*L. agilis* DSM 20509	Agilicin (Agl)	MSDK-MENKKKLTTADLAKVTGG	SRYYGNGITCG-KHKCTVNWGQAWTCGVNRLANFGH–G————–NC—	56.76
*L. ruminis* DSM 20403	Ruminicin (Rum)	—————MRQLSEKELKKIMGG	–KYYGNGVYCG-KHKCRVDWGQAWGCSVNRWGAAVGTGGKATIGHC—	54.55
*L. aquaticus* DSM 21051	Aquaticin (Aqu)	———————————-MNGG	–KNYGNGVYCTKKHGYKVDWGQAWSIIGNNSAANSTTRGAAGWKSK—-	47.73
*P. pentosaceus* DSM 20336	Penocin A (PenA)	———-MTEIKVLNDKELKNVVGG	–KYYGNGVHCG-KKTCYVDWGQATASIGKIIVNGWTQHGPWAHR———-	47.62
*L. futsaii* JCM 17355	Futcin (Fut)	MKGRYVNMKKVIDENSLSLISGG	–KYYGNGVSCG-KHTCKVNWGQAWNESVNRWGNSWVNGLTGLRQH—	44.19
*L. acidipiscis* DSM 15836	Acidicin (Acd)	———————–LSLEESSSVIGG	—-KYYGNGLHIPKHGKPYINWGQAIQSIGKISYHGWVNGITSGAAGVGRH	29.55

**Figure 1:**
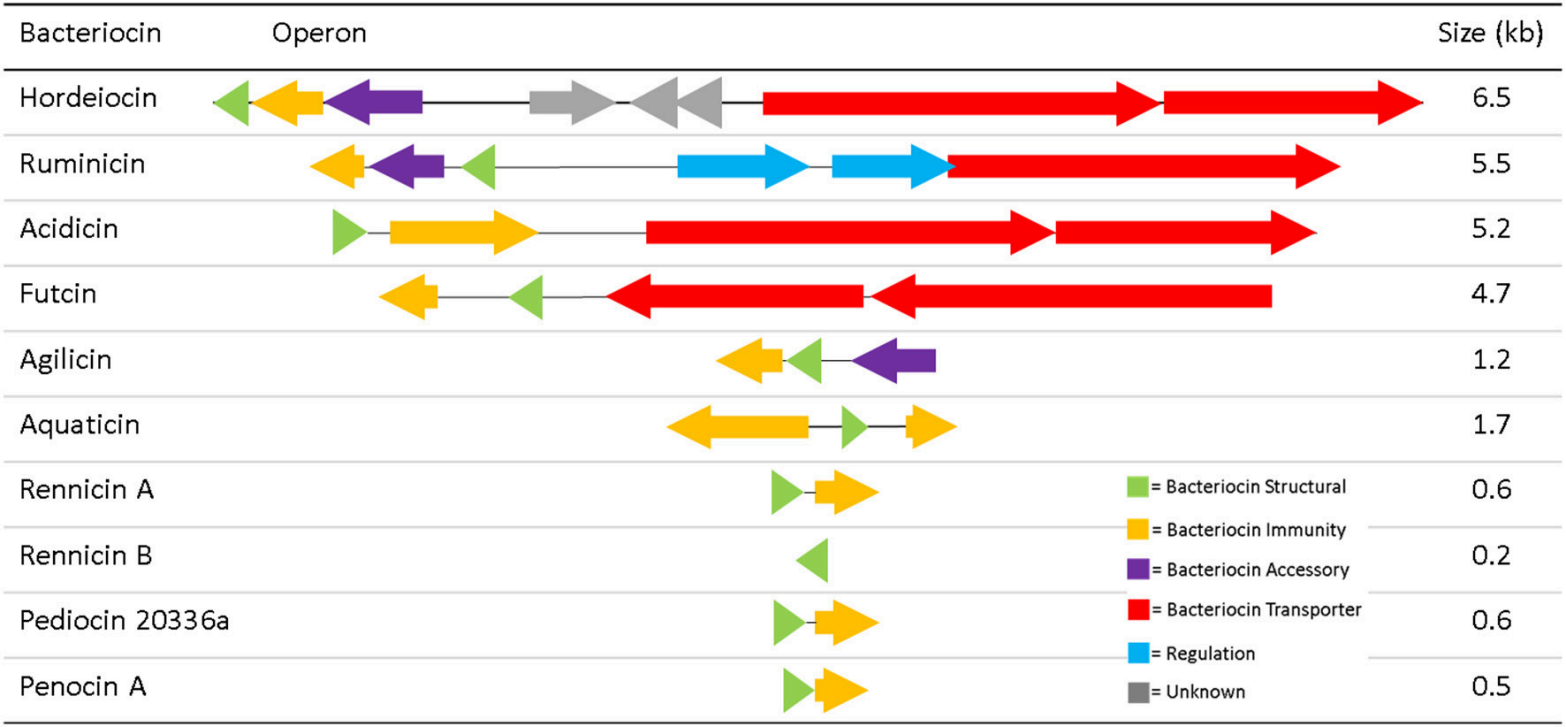
Class IIa Bacteriocin Operons.

Typically at least four genes are commonly required for the production of a class II bacteriocin; a structural gene, a transporter, an immunity gene and a gene encoding an accessory protein (Drider et al., 2006). The operons for the ten “silent” class IIa bacteriocins differed in their composition, with several lacking the genes for some of the necessary bacteriocin associated components (Figure 1). Each of these nine novel bacteriocins were named based on the species which produced them; e.g., ruminicin produced by *L. ruminus*. Of the operons identified, only those encoding hordeiocin and ruminicin were found to encode all four key components for bacteriocin production. The acidicin operon also appeared to be complete as the structural peptide doesn't contain any cysteine residues for disulphide bond formation, thus the accessory protein is probably unnecessary. The futcin operon was the only other such operon which encoded an ABC transporter for bacteriocin transport and leader cleavage. The remaining operons were composed of two to three genes encoding just the structural gene and immunity protein, with the agilicin operon also encoding an accessory protein.

### Heterologous cloning

As there was no identifiable bacteriocin production from these eight strains, it was decided to express these novel bacteriocins first in an *E. coli* host. A modified pETcoco^TM^-2 vector, pMPB1, generated by Mesa-Pereira et al. (2017) was used for bacteriocin expression. It was previously found that only the structural gene and the ABC transporter are required to express pediocin PA-1 using this system. As over half of the bacteriocins identified here lack an associated ABC transporter, the pediocin PA-1 transporter PedD was used for the expression of each of these bacteriocins. PedD contains an intrinsic domain for cleavage of the bacteriocin leader sequence, and as these transporters rely on this leader sequence for peptide processing each of these bacteriocins were cloned separately using both their native leader sequence and as a fusion protein containing the pediocin PA-1 leader. Recursive PCR was used to generate the genes for each of these fusion proteins; genes for the natural bacteriocins and the PedD transporter were amplified by PCR (Prodromou and Pearl, 1992). An In-Fusion reaction was then used to assemble and join these PCR products to the enzyme-digested pMPB1 vector. For production in *L. paracasei* NFBC338, a pNZ44-derived vector was used; in this case the bacteriocin was cloned as a fusion with the pediocin leader along with the PedD transporter. PCR products for these reactions were obtained using positive pMPB1 plasmids as a template. In-Fusion reaction was then used to assemble and join these PCR products to the enzyme-digested pNZ44 vector. The vectors used are shown in Supplementary Table 1.

### Production

*E. coli* Tuner^TM^ (DE3) cells transformed with the newly constructed plasmids were grown in LB supplemented with glucose to maintain a low-copy number and bacteriocin expression was induced by co-incubation of the clones with 50 μM IPTG for 3 h. WDAs were used to identify bacteriocin activity from the bacterial supernatants and cell lysates. Bacteriocins were exclusively found in the bacterial supernatant. Antimicrobial activity was lower in cases where the native leader of the bacteriocin sequence was used; with only four of the nine strains displaying zones of inhibition against a sensitive indicator (pediocin 20336a has an identical leader to pediocin PA-1). For expression in *L. paracasei* NFBC338, cells were transformed by electroporation with the bacteriocin-containing pNZ44 vectors. Positive transformants were grown overnight and the neutralized cell free supernatant was used to identify bacteriocin production in WDAs.

Of the bacteriocins expressed with the pediocin PA-1 leader sequence in *E. coli*, 8 of the ten displayed anti-microbial activity from the cellular supernatant against at least one indicator organism (Table 2). Purification of the bacteriocin peptides from the *E. coli* bacterial supernatant and MALDI-TOF mass spectrometry were used to determine the mass of the active peptides. These masses correlated closely with the predicted mass of the bacteriocin structural peptide, allowing us to identify the bacteriocin produced. This approach confirmed the production of nine of the ten bacteriocins, aquaticin was not identified using these methods, potentially due to low levels of production by the cells (Supplementary Figure 1). The percentage amino acid identity of these bacteriocins in comparison to pediocin ranges from 29.5% to 93.2%. Production levels of bacteriocins were shown to vary between the *E. coli* and *L. paracasei* expression systems. *E. coli* transformants actually displayed greater or equal bacteriocin activity compared to those produced by *L. paracasei* (Table 3). The only exceptions were the original pediocin PA-1 which was used as a control and rennicin B which was produced in very low quantities by the *E. coli* producer where production was only noted after purification of the bacteriocin from the cellular supernatant. Interestingly, *L. paracasei* NFBC338 failed to produce futcin which was produced by *E. coli*.

**Table 2:** Spectrum of bacteriocin activity.

**Species**	**Strain**	**PedA**	**Acd**	**RenB**	**Hrd**	**Ped 20336**	**Aqu**	**RenA**	**PenA**	**Fut**	**Rum**	**Agl**
*Bacillus cereus*	DPC 6087											
*Enterococcus faecalis*	LMG 7397	4.33 ± 0.47			2.67 ± 0.47	4 ± 0		3 ± 0	0.67 ± 0.47	1.67 ± 0.47	1.17 ± 0.24	2.17 ± 0.24
*E. faecium*	DPC 4898											
*E. saccharolyticus*	DPC 4902	6 ± 0			3.67 ± 0.47	5.67 ± 0.47		2.33 ± 0.47	1.33 ± 0.94	2 ± 0	2 ± 0	3.33 ± 0.47
*E.mundtii*	LMG 10748	5 ± 0			2.67 ± 0.47	4.67 ± 0.47		3.33 ± 0.47	1.33 ± 0.94	1.83 ± 0.24	2 ± 0	3 ± 0
*Lactobacillus ingluvei*	DSM 15946											
*L. amylovorus*	DSM 20531										0.67	
*L. apodemi*	DSM 16654	3.33 ± 0.47			2.67 ± 0.47	3.67 ± 0.47		0.67 ± 0.47		0.33 ± 0.47	1.67 ± 0.47	2.67 ± 0.47
*L. bulgaricus*	LMG 6901											
*L. crustorum*	JCM 15951	0.33 ± 0.47			1 ± 0	1 ± 0					0.33 ± 0.47	0.33 ± 0.47
*L. delbrueckii indicus*	DSM 15996											
*L. delbrueckii lactis*	LMG 7942											
*L. farraginis*	DSM 18382											
*L. amylophilus*	DSM 20509				2.50 ± 0.41	0.33 ± 0.47			0.67 ± 0.47		1.33 ± 0.47	
*L. intestinalis*	DSM 6629											
*L. kimchicus*	JCM 15530				3 ± 0	0.33 ± 0.47						
*L. mali*	DSM 20444	7.67 ± 0.47	1.50 ± 0.41		4.33 ± 0.47	6.33 ± 0.47		1 ± 0	1.50 ± 1.08	2.17 ± 0.85	2 ± 0	4.50 ± 0.41
*L. nodensis*	DSM 16982				3.67 ± 0.47	1.67 ± 0.47						3.50 ± 0.71
*L. paralimentarius*	DSM 13961	0.67 ± 0.47			1 ± 0.47	1 ± 0.47						
*L. plantarum*	DSM 13273	0.33 ± 0.47			1.33 ± 0.47	1.33 ± 0.47						0.33 ± 0.47
*L. salivarius*	DPC 6502											
*Leuconostoc fallax*	DSM 20189											
*Listeria innocua*	DPC 3572	8 ± 0			4.67 ± 0.47	7.33 ± 0.47		6.33 ± 0.94	2.50 ± 0.41	2.00 ± 0	3.33 ± 0.47	5 ± 0
*Li. monocytogenes*	DPC 6893	3 ± 0			2 ± 0	2.67 ± 0.47		2 ± 0	0.67 ± 0.47		1 ± 0	2 ± 0
*Li. monocytogenes*	DPC 6894	5 ± 0			4 ± 0	4.33 ± 0.47		2 ± 1.41	0.67 ± 0.47	0.83 ± 0.24	2 ± 0	4 ± 0
*Li. monocytogenes*	WSL 1416	3.33 ± 0.94			2 ± 0.82	3.33 ± 0.94		2.67 ± 0.47	0.67 ± 0.47	1 ± 0	1 ± 0	2 ± 0.82
*Pseudomonas aeruginosa*	APC 2064											
*Pediococcus stilesii*	DSM 18001	1 ± 0			1 ± 0	1 ± 0			0.67 ± 0.47			
*P. ethanolidurans*	DSM 22301	5.67 ± 0.47			3.67 ± 0.47	5.67 ± 0.47		1.50 ± 0.41	2 ± 1.41	3 ± 0	2.17 ± 0.24	3.33 ± 0.47
*P. clausenii*	DSM 14800				2 ± 0	0.67 ± 0.47			1 ± 0.82		1 ± 0	0.67 ± 0.47
*Staphylococcus aureus*	C55											
*Salmonella typhimurium*	LT2											
Total no. of strains inhibited		14	1	0	18	18	0	10	12	9	14	14

*Antimicrobial activity is calculated as the radius of the zone of inhibition from a well diffusion assay measured in millimeters. PedA, pediocin PA-1. Acd, acidicin. RenB, rennicin B. Hrd, hordeiocin. Ped 20336, pediocin 20336a. Aqu, aquaticin. RenA, rennicin A. PenA, penocin A. Fut, futcin. Rum, ruminicin. Agl, agilicin*.

**Table 3:** Bacteriocin Activity (BU/ml) vs. *L. innocua* DPC3572.

	***E. coli* Tuner (DE3)**	***L. paracasei* NFBC338**
Pediocin PA-1 (PedA)	+	++
Rennicin A (RenA)	+	+
Ruminicin (Rum)	+++	+++
Agilicin (Agl)	++	++
Aquaticin (Aqu)	–	–
Rennicin B (RenB)	–	+
Acidicin (Acd)	–	–
Pediocin 20336a (Ped20336)	+	+
Futcin (Fut)	+	–
Hordeiocin (Hrd)	+++	++
Penocin A (PenA)	++	+

*Bacteriocin units per ml (BU/ml) were calculated as the inverse of the highest dilution of bacterial supernatant showing activity against L. innocua DPC3572 in a well diffusion assay. + = 0 – 160 BU/ml, ++ = 320 – 2560 BU/ml, +++ = 5120 – 10240 BU/ml*.

### Spectrum of activity

The spectrum of activity was determined using WDAs against 32 indicator strains for bacteriocins produced by the *E. coli* transformants encoding the pediocin leader (Table 2). Despite being of the same class of bacteriocins, these novel antimicrobials display a varying spectrum of activity. Interestingly, pediocin 20336a and hordeiocin inhibited the growth of a larger number of the indicators tested than pediocin PA-1. Aquaticin and rennicin B failed to display antimicrobial activity in the crude supernatant from the *E. coli* heterologous host, whilst acidicin displayed activity exclusively against *L. mali* in this assay. Many of these bacteriocins, however, can inhibit a wide range of Gram positive bacteria including potential pathogens such as *Enterococcus faecalis*. As with most class IIa bacteriocins, these also display potent anti-listerial activity. Interestingly, *L. mali* appears to also be an extremely sensitive indicator for testing the activity of class IIa bacteriocins here and may prove a safer alternative over the use of *Listeria* strains to test the activity of these bacteriocins.

### Bacteriocin structures

Class IIa bacteriocins tend to have a relatively conserved structure. However, some of these novel bacteriocins display key differences in the typical conserved regions associated with previously characterized class IIa bacteriocins. A three-stranded β-sheet structure can be found at the N-terminus, this is often stabilized by a conserved disulphide bridge formed between two cysteine residues at the N-terminus. Because aquaticin has only a single cysteine residue and acidicin completely lacks cysteine residues these bacteriocins would be unable to produce the conserved disulphide bond. The C-terminus is less conserved and can be composed of one or two α-helices and an elongated C-terminal tail which can fold back on the α-helix, forming a hairpin-like structure. A C-terminal disulphide bond can stabilize this hairpin structure in certain bacteriocins and pediocin 20336a, rennicin A, rennicin B, hordeiocin, agilicin and ruminicin all have the ability to form this disulphide bond (Fimland et al., 2005).

The N-terminal “YGNGV/L” region is highly conserved in these peptides, interestingly agilicin is the only such bacteriocin where the valine or leucine residue in this sequence has been replaced by an isoleucine. These peptides also contain a conserved hinge region (VD/NWGXA) which separates the N-terminal β sheet configuration from the C-terminal α helix (Uteng et al., 2003). Acidicin contains a valine to isoleucine substitution in this motif, a modification only previously seen in lactococcin MMFII (Ferchichi et al., 2001).

## Discussion

Due to the growing availability of genomic data and the improvement of software a growing number of novel bacteriocins are being identified (Letzel et al., 2014; Zhao and Kuipers, 2016). There continues however, to be a disconnect between the identification of these genes and the actual production of these bacteriocins *in vitro*. Whilst metagenomic data provides the ability to detect these genes, this is often not correlated to the isolation and characterization of the producing strain. Thus, whilst bacteriocin genes are being discovered at a much greater rate from metagenomic data, the isolation of these antimicrobials themselves has proved more difficult.

One method to bridge the gap between the discovery of bacteriocin genes and their *in vitro* production is to heterologously express these genes in a new host. This could prove particularly valuable for strains that are non-culturable or that are extremely difficult to grow in the laboratory. Developing and optimizing cloning techniques for individual bacteriocins requires time, which can make it a laborious task when working with a large number of potential bacteriocin genes identified in a genomic screen. The methods used by Mesa-Pereira et al. (2017) and in this study provide a rapid mechanism to express non-lantibiotic bacteriocins such as class IIa bacteriocins and class IId bacteriocins. It has been determined that the presence of the bacteriocin structural gene and the bacteriocin transporter is sufficient to express these bacteriocins using this system (Mesa-Pereira et al., 2017). This provides a quick and easy method to produce such bacteriocins, even if the original operons identified are incomplete. Using this method, it was possible to express novel bacteriocins identified from *in silico* screening of the *Lactobacillus* Genus Complex, despite many of these lacking the obligatory genes required for bacteriocin production by the parent strains. It is unclear as to why such strains encode intact bacteriocin and immunity genes whilst lacking the machinery needed to actually produce and secrete these antimicrobials. One potential explanation for this is that these strains may have maintained the bacteriocin immunity gene under selective pressures and that the neighboring small bacteriocin encoding gene may also have been maintained whilst the larger transport machinery was lost.

The bacteriocin expression system used is based on the pediocin PA-1 operon, using the associated transporter PedD to transport the expressed bacteriocin from the cell. Each of the ten bacteriocins described here was cloned in an *E. coli* heterologous host alongside this transporter using both its native leader sequence and as a fusion containing the pediocin PA-1 leader as opposed to its own. Of the ten bacteriocins studied here, nine were novel. The bacteriocins varied from 93.2% to 29.5% amino acid identity to pediocin PA-1. Nine of the ten bacteriocins displayed antimicrobial activity (aquaticin activity was not seen and the peptide was not identified after purification and MALDI-TOF mass spectrometry); the production of these bacteriocins shows the flexibility in the PedD transporter and its ability to secrete several bacteriocins. Production levels of these bacteriocins in an *E. coli* host was greater when they were expressed with the pediocin PA-1 leader sequence rather than their own, this is not surprising given that PedD has evolved to cleave the pediocin PA-1 leader. Four bacteriocins were produced and secreted using their native leader which reflects a degree of redundancy in the specificity of the cleavage domain in the PedD transporter.

The bacteriocins produced displayed a varying spectrum of activity despite all belonging to the class IIa bacteriocins. Pediocin PA-1 is an important commercial additive used in food production in the form of powdered fermentates such as ALTA® 2351 (Kerry Bioscience) for the inhibition of *Listeria* species as well as other food spoilage and pathogenic bacteria. The discovery of novel bacteriocins here with a greater inhibitory range indicates that alternative bacteriocins may prove to be more effective additives in food; it also opens up the possibility for extending the use of these bacteriocins for alternative applications such as potential therapeutic uses. Further studies into the effect the structure of these bacteriocins can have on bacteriocin activity may also allow for targeted peptide engineering of these bacteriocins to improve activity and extend their range of inhibition in the future.

The structure of these bacteriocins can be affected by differences in sequences of these peptides. The N-terminal β-sheet structure of these bacteriocins can be stabilized by the presence of a disulphide bridge, aquaticin and acidicin, however, lack the ability to form such a bond. Sit et al. (2012) previously found that this disulphide bridge can be removed from class IIa bacteriocins; this reduces but does not eliminate the peptides' inhibitory activity. This may explain the lower levels of activity seen for aquaticin and acidicin. The hydrophobic/amphiphilic C-terminus of these peptides is less conserved than the N-terminus and is involved in membrane insertion which results in pore formation thus killing the target cell and also determines the spectrum of activity of the bacteriocins (Johnsen et al., 2005). The C-terminus is composed of an α-helix followed by a C-terminal tail which forms a hairpin and folds back upon the α-helix. This motif can be stabilized by the presence of a C-terminal disulphide bridge which makes the structure less flexible. Class IIa bacteriocins lacking this second disulphide bridge tend to be more heat sensitive and can undergo unfolding, making them less active at 37°C (Kaur et al., 2004). Acidicin, futcin, aquaticin and penocin A all lack the ability to form this disulphide bridge, which may explain the lower levels of activity seen for these bacteriocins as the majority of the indicator organisms used here are grown at 37°C. In certain bacteriocins, which lack the ability to form this disulphide bridge, the interaction between tryptophan residues found just after the hinge region and at the C-terminus can stabilize the hairpin fold (Fimland et al., 2002). Penocin A and aquaticin both have terminal tryptophan residues which would compensate for an absent disulphide bond. Futcin has a tryptophan residue at position 33 in the mature peptide; however this is not predicted to be involved in the stabilization of the hairpin fold; acidicin lacks a stabilizing terminal tryptophan residue altogether. This may suggest that such an extended hairpin structure does not form in these bacteriocins. Structural differences between these bacteriocins may not only affect their inhibitory activity but also may affect the ability of the pediocin transporter to secrete these bacteriocins. The bacteriocins which display the greatest divergence form pediocin PA-1 were, however, shown to be secreted here indicating a level of redundancy in the transporter which may extend to the production of other unmodified bacteriocins. This potential extension of the classes of bacteriocins secreted by this system is supported by the activity of the transported EnkT from *E. faecium* NKR-5-3 which is involved in the transport of a class IIa bacteriocin, two peptides of a class IIb bacteriocin and also an inducer peptide (Sushida et al., 2018). This emphasizes the potential flexibility of these bacteriocin ABC transporters.

The novel bacteriocins described here cluster into different groups upon alignment. Pediocin, pediocin 20336a, rennicin A and rennicin B display between 84% and 93% homology to each other. Despite being 90.9% identical to pediocin PA-1, rennicin B did not display activity in the crude supernatant from the *E. coli* heterologous host; it did however display equal levels of activity compared to rennicin A when expressed in *L. paracasei* NFBC338. Two amino acid substitutions in rennicin B may explain this as they occur in important structural regions for the bacteriocin. The Gly29-Ser29 substitution is found in the C-terminal α-helix of the peptide which is involved in membrane insertion. The substitution of a non-polar amino acid for a larger polar one here may affect the formation of the helix and membrane insertion. A Gly36-Ser36 substitution occurs in a double glycine motif which follows the α-helix. This motif may provide the flexibility for the C-terminal tail to fold back upon the helix (Fimland et al., 2002), this flexibility may be lost due to the substitution with a larger serine residue.

Hordeiocin, agilicin, futcin and ruminicin to a lesser extent, also cluster together, displaying between 60% and 76% amino acid identity. Hordeiocin, agilicin and ruminicin all display a relatively broad spectrum of activity (inhibiting between 18 and 14 strains), whilst futcin is more narrow spectrum (inhibiting nine strains) which again may be due to a lack of a C-terminal stabilizing disulphide bridge. Penocin A, acidicin and aquaticin lack a high degree of similarity to each other and the other bacteriocins produced.

Through analysis of the data from previous *in silico* bacteriocin screens there is the potential to use these cloning systems to a far greater extent to increase the current repertoire of unmodified class II bacteriocins. Whilst this study focuses on the *Lactobacillus* Genus Complex, numerous other studies have analyzed large amounts of genomic data from other sources to identify novel bacteriocins. Zheng et al. (2015) screened 700 shotgun metagenomic datasets from the Human Microbiome Project for the presence of bacteriocin operons. Of the 4875 putative bacteriocin genes found here, there were 3048 potential class II bacteriocins including 50 class IIa pediocin homologs, all of which represent potential candidates for use in this expression system. Similarly Alvarez-Sieiro et al. (2016) screened 238 genomes of lactic acid bacteria using BAGEL3, from this they identified 785 putative bacteriocin genes of which 514 encoded potential unmodified bacteriocin genes containing 31 class IIa homologs. Other such *in silico* screening studies have identified 209 further unmodified bacteriocin genes (Kjos et al., 2011; Azevedo et al., 2015; Walsh et al., 2015; Liu et al., 2016). While duplications and false positives are likely to occur in these datasets, even if a small proportion of these genes can be analyzed using this expression system it represents a significant extension of the class II bacteriocins.

Thus whilst *in silico* genomic studies can lead to the identification of bacteriocins, often this research is not carried forward leading to the characterization of these antimicrobials. The simple system used here outlines how bacteriocin genes identified through *in silico* screening of the *Lactobacillus* Genus Complex could easily be heterologously expressed. Bacteriocins which otherwise would not have been produced by the original strain due to tight regulation of the operon or loss of necessary genes were able to be produced and studied. Ten class IIa bacteriocins were studied here, nine of which were novel. Nine of these bacteriocins were produced and secreted by the PedD transporter, despite showing less than 30% identity to pediocin PA-1, which reflects the permissiveness of the transporter in secreting these peptides. These novel bacteriocins notably extend the group of class IIa bacteriocins, however these would likely not have been produced and analyzed if not for the expression systems used here. This has allowed us to reincarnate these bacteriocin relics, and provides the capacity to identify and produce a vast range of novel bacteriocins identified by other *in silico* screens which may otherwise be destined to remain uncharacterized in their genomic graveyard.

## Author contributions

FC, BM-P, MR, CH, and RPR conceived and designed the study. FC, BM-P, and PO carried out the experimental work and analysis. All parties contributed to the preparation of the manuscript.

### Conflict of interest statement

The authors declare that the research was conducted in the absence of any commercial or financial relationships that could be construed as a potential conflict of interest.
