# Supplementary material for: Reincarnation of Bacteriocins From the Lactobacillus Pangenomic Graveyard
Source: Front Microbiol. 2018 Jul 2;9:1298. doi: 10.3389/fmicb.2018.01298 (PMC6036575; doi:10.3389/fmicb.2018.01298)

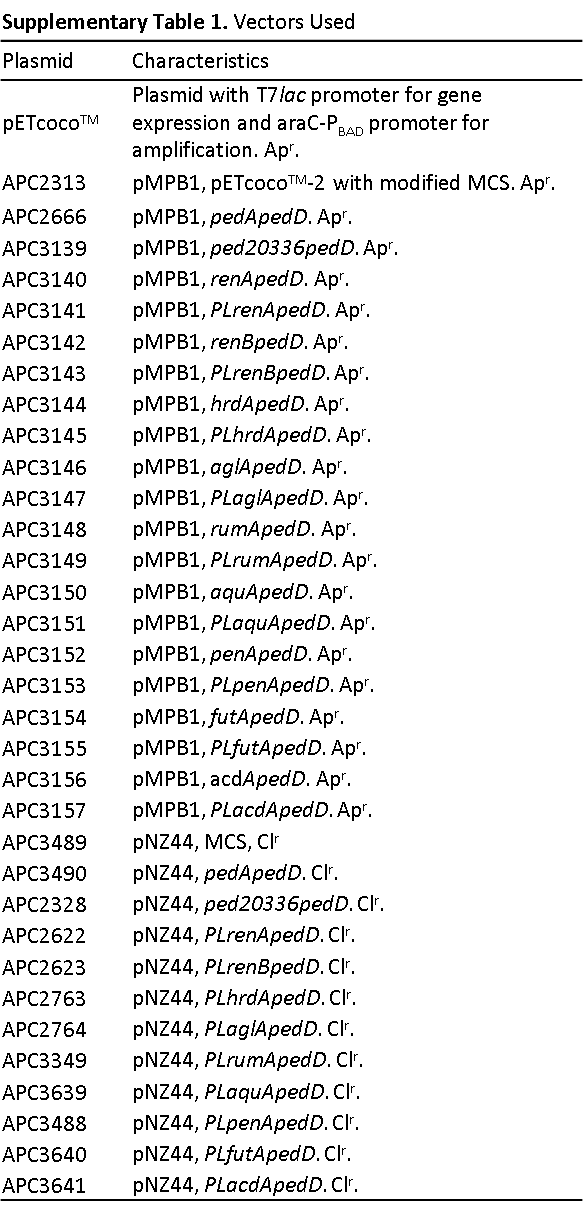


*MCS, multiple cloning site. Ap^r^, ampicillin resistant. Cl^r^, chloramphenicol resistance. *PL*, pediocin PA-1 leader sequence fusion gene.


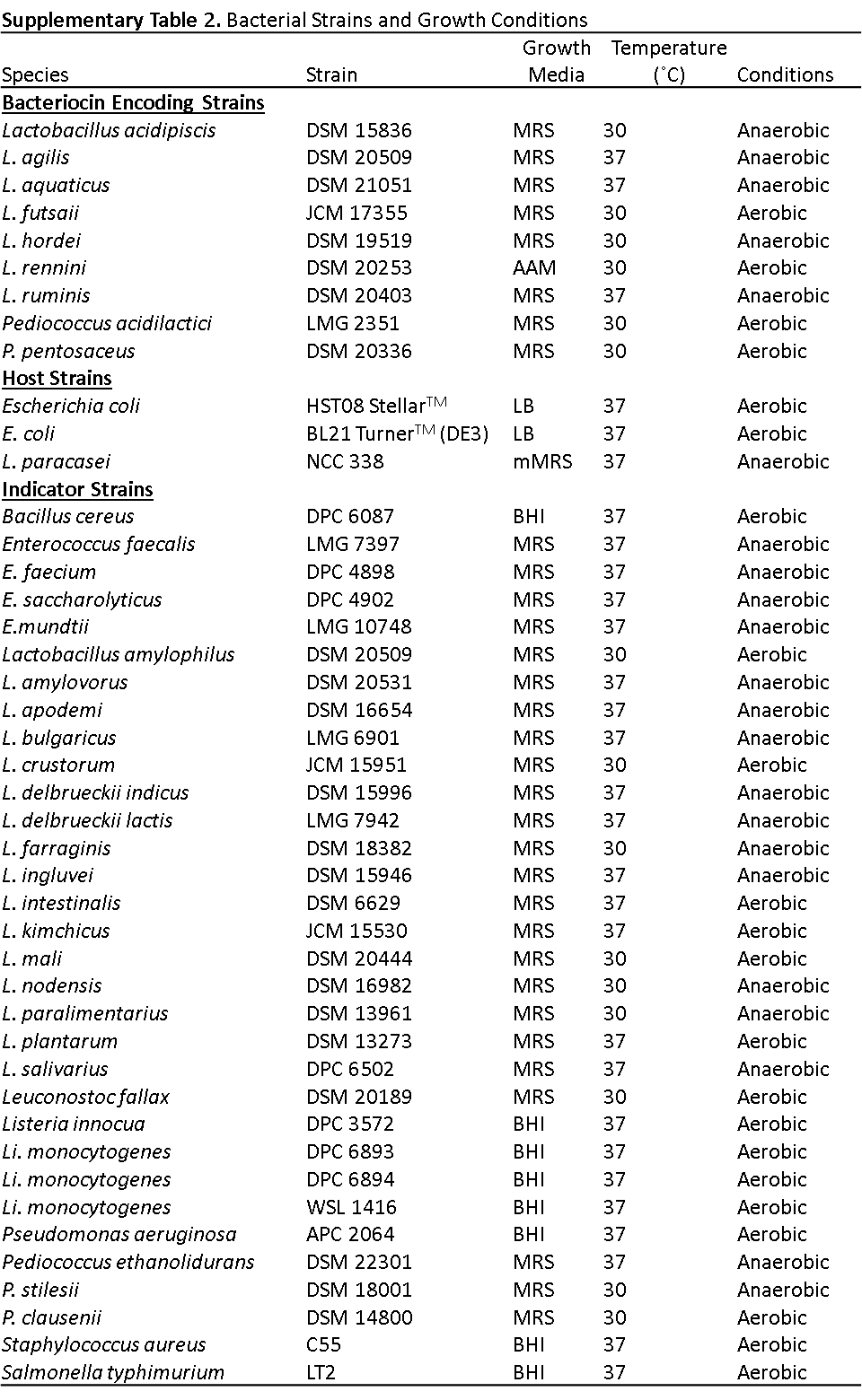


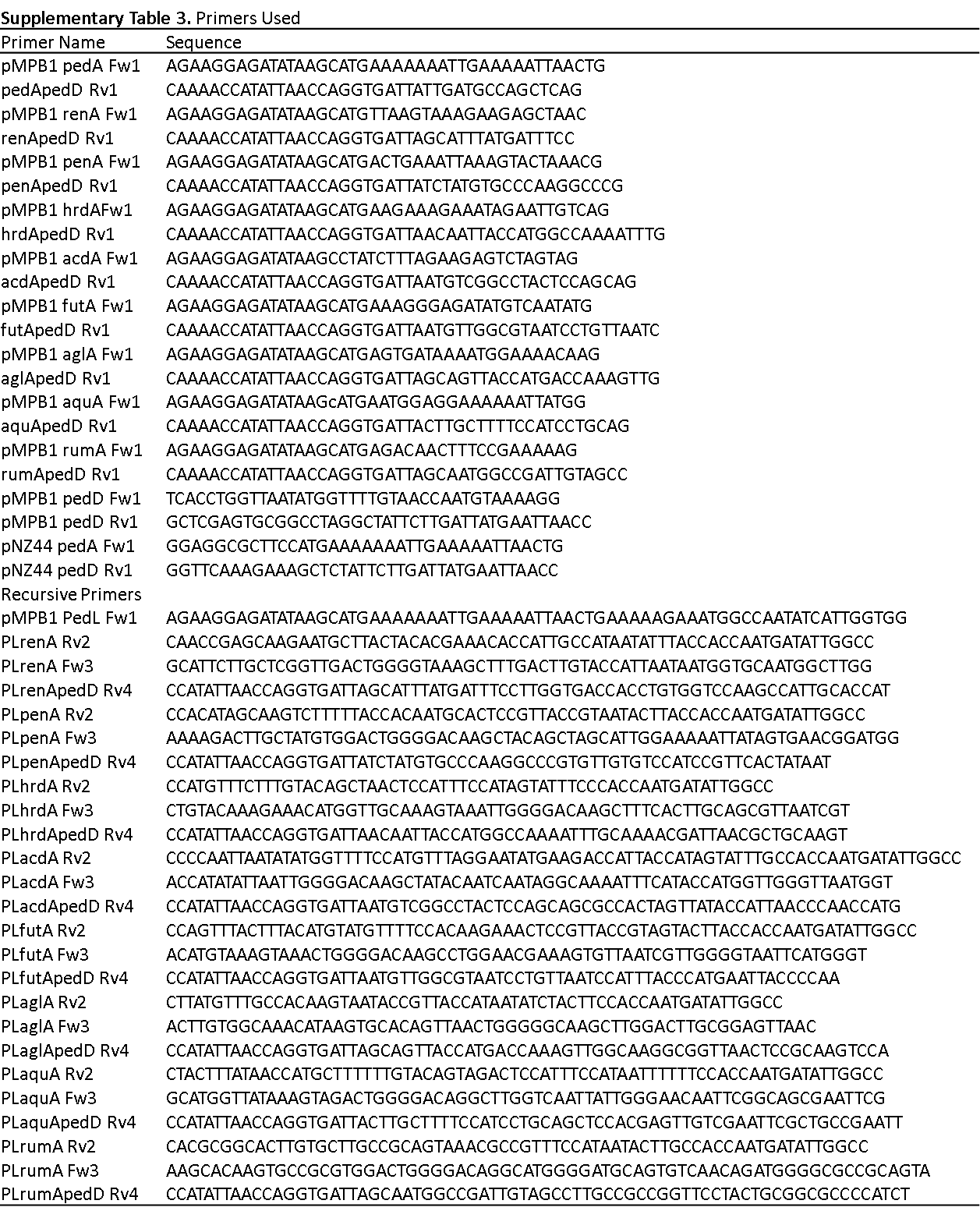

Supplement: Supplementary file 1 [file Data_Sheet_1.docx]
