# Supplementary figures and images for: Reincarnation of Bacteriocins From the Lactobacillus Pangenomic Graveyard
Source: Front Microbiol. 2018 Jul 2;9:1298. doi: 10.3389/fmicb.2018.01298 (PMC6036575; doi:10.3389/fmicb.2018.01298)

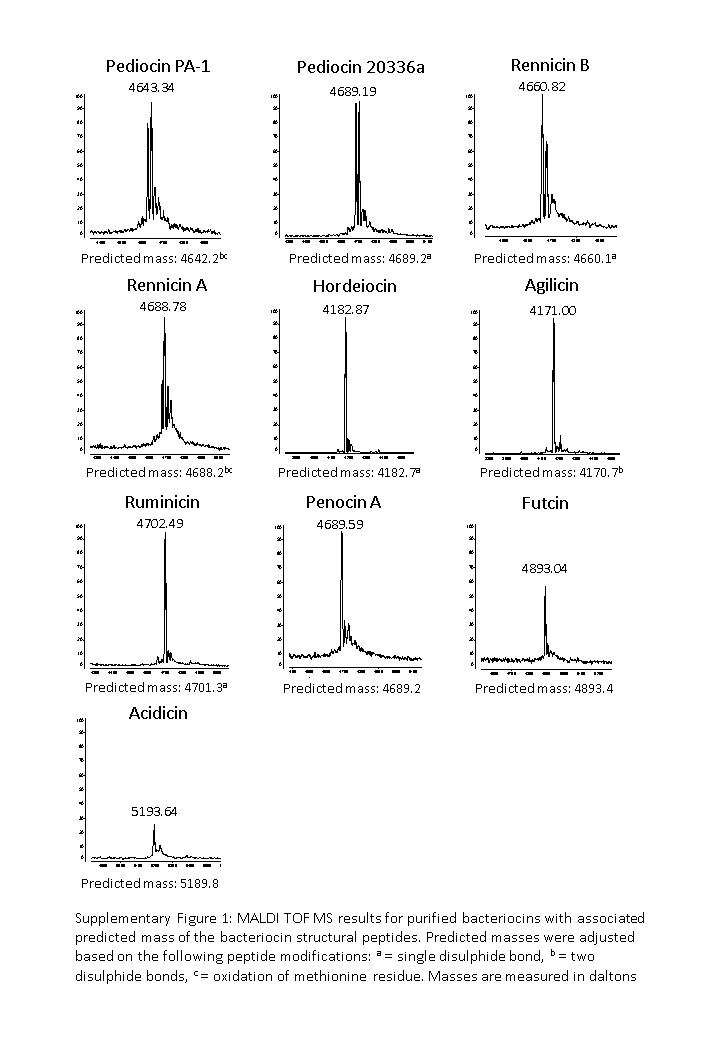

Supplement: Supplementary file 2 [file Image_1.TIF]
